# Identification of potential target genes of the trematode transport RNA-derived small RNAs among differentially expressed genes of human monocytes

**DOI:** 10.18699/vjgb-26-84

**Published:** 2026-09

**Authors:** E.A. Lishai, M.Y. Pakharukova

**Affiliations:** Institute of Cytology and Genetics of the Siberian Branch of the Russian Academy of Sciences, Novosibirsk, Russia Novosibirsk State University, Novosibirsk, Russia; Institute of Cytology and Genetics of the Siberian Branch of the Russian Academy of Sciences, Novosibirsk, Russia Novosibirsk State University, Novosibirsk, Russia

**Keywords:** opisthorchiasis, tRNA, exosome-like vesicles, RNA interference, transcriptomics, human monocytes, описторхоз, тРНК, экзосомо-подобные везикулы, РНК-интерференция, транскриптомика, моноциты человека

## Abstract

RNA interference is an evolutionarily conserved mechanism of post-transcriptional gene expression regulation present in all living organisms. It is highly relevant to multiple areas of molecular medicine, including diagnostics and novel immunomodulatory approaches. Recent studies have increasingly highlighted the role of extracellular vesicles and their cargo in parasite-host interactions. The trematode Opisthorchis felineus, which parasitizes the human biliary tract, causes opisthorchiasis, a disease associated with chronic inflammation and other hepatobiliary injuries. The long-term survival of the parasite is likely linked to modulation of host immune response. Nevertheless, the mechanism by which liver fluke vesicles affect human immune cells remains unclear. The aim of this study was to identify potential human gene targets of tRNA-derived small RNAs and to assess their enrichment among differentially expressed genes (DEGs) in the transcriptome of THP-1 human monocytes. Target prediction for eight most abundant O. felineus tRNA fragments identified 1,299 potential target genes in human monocytes. Seven cDNA libraries prepared from THP-1 monocytes before and after treatment with vesicles from adult O. felineus were sequenced using paired-end 2 × 150 bp sequencing (DNBseq, BGI), yielding 43.8 million reads per library. Among 1484 DEGs, 159 predicted targets of tRNA-derived small RNAs showed significant expression changes upon vesicle treatment. These genes were associated with pathways involved in cell-cycle regulation and cell death, including DNA integrity control (M16357), G1/S transition (M2074), apoptosis (M15303), and programmed cell death (M27436), as well as immune-related pathways, including the monocyte pathway (M4956), mononuclear macrophage differentiation (M25144), MHC-I antigen presentation (M1066), and proteasome-mediated antigen processing (M1070). These findings suggests that O. felineus tRNA-derived small RNAs may contribute to the regulation of these processes in human monocytes.

## Abstract (ru)

РНК-интерференция – эволюционно консервативный механизм посттранскрипционной регуляции генов, представленный у всех видов живых организмов, в настоящее время актуален для многих направлений молекулярной медицины, включая диагностику и новые подходы к иммуномодуляции. В последнее время появляется все больше данных об участии внеклеточных везикул и их компонентов в контексте взаимодействия между паразитом и хозяином. Трематода Opisthorchis felineus, паразитирующая в желчных протоках человека, вызывает описторхоз, что сопровождается хроническим воспалением и рядом неблагоприятных последствий. Длительное выживание паразита, вероятно, связано с иммуномодуляцией хозяина. Однако механизм влияния везикул трематод на моноциты человека неизвестен. Целью настоящей работы было найти потенциальные гены-мишени фрагментов тРНК трематод в геноме человека и проанализировать их представленность среди дифференциально экспрессирующихся генов (ДЭГ) в транскриптоме моноцитов человека линии THP-1. В результате поиска генов-мишеней для восьми наиболее представленных фрагментов тРНК O. felineus было выявлено 1299 потенциальных генов, которые могут быть мишенями в моноцитах человека. Семь кДНК библиотек клеточной культуры моноцитов человека линии THP-1 до и после обработки выделенными везикулами взрослой особи O. felineus были просеквенированы методом парных прочтений 2 × 150 пар оснований (технология DNBseq, BGI, Китай), получено около 43.8 млн прочтений на библиотеку. Среди 1484 ДЭГ в моноцитах было обнаружено 159 генов-мишеней фрагментов тРНК везикул, экспрессия которых значимо менялась при обработке экзосомо- подобных везикул (ЭПВ). Выявленные потенциальные гены-мишени относились к путям, связанным с регуляцией клеточного цикла и клеточной гибели (контроль целостности ДНК (M16357), переход из фазы G1 в фазу S (M2074), апоптоз (M15303) и программируемая клеточная гибель (M27436)), иммунного ответа (путь моноцитов (M4956), дифференцировка мононуклеарных макрофагов (M25144), презентация антигена через MHC-1 (M1066), процессинг антигена через протеасому (M1070)). Вероятно, фрагменты тРНК трематоды O. felineus могут вносить вклад в регуляцию этих процессов в моноцитах хозяина.

## Introduction

RNA interference is an evolutionarily conserved and widely distributed mechanism of post-transcriptional gene regulation across living organisms. Increasing interest in this field of genetics and molecular biology reflects the growing potential of non-coding RNAs for regulating target gene expression, supporting diagnostics, and enabling novel immunomodulatory strategies. Many small non-coding RNAs that repress target gene expression at the post-transcriptional level are transported within extracellular vesicles. However, the contribution of small RNAs to interspecies communication, including parasite–host interactions, remains incompletely understood. A relevant model for studying parasite RNA–host interactions is the epidemiologically important trematode Opisthorchis felineus (Rivolta, 1884).

O. felineus parasitises the bile ducts of fish-eating mammals, including humans. Chronic infection is associated with persistent inflammation and biliary intraepithelial neoplasia (Xia et al., 2015; Mordvinov et al., 2021; Lishai et al., 2024). The long-term survival of trematodes within a host is likely due to their ability to manipulate the host’s immune system. Consistent with this, helminth infections have been associated with attenuated manifestations of autoimmune diseases, such as Crohn’s disease and asthma (Araújo et al., 2004; Saltykova et al., 2014).

Immune modulation, as well as parasite-driven effects on other host processes, including carcinogenesis, requires active communication between the parasite and the host. One mechanism that may mediate such communication involves extracellular vesicles that are internalised by host cells through distinct endocytic pathways. For example, human cholangiocytes internalise O. felineus exosome-like vesicles (ELVs) via clathrin-dependent endocytosis (Pakharukova et al, 2023). These ELVs carry diverse bioactive molecules, including small non-coding RNAs, such as tRNA-derived small RNAs

tRNA-derived small RNAs (tsRNAs), generated from precursor or mature tRNAs, are increasingly recognised as a novel class of regulatory molecules implicated in various diseases (Prehn, Jirström, 2020; Chen et al., 2024; Strømme et al., 2024). tsRNAs have been detected in extracellular vesicles, including exosome-like vesicles secreted by multiple parasites, and may modulate host cell functions (Artuyants et al., 2020; Fontenla et al., 2022; Kusakisako et al., 2023).

Monocytes are key cells of the innate immune system and participate in the early response to invading pathogens (Passos et al, 2017). Parasite-derived extracellular vesicles have been shown to affect monocyte polarisation and cytokine production in the host (Silverman et al., 2010; Lertjuthaporn et al., 2022; Norouzi et al., 2022; Khowawisetsut et al., 2023). Nevertheless, the responses of human monocytes to ELVs released by O. felineus remain poorly characterised. Furthermore, it is unknown whether tsRNAs transported by O. felineus ELVs can directly influence human monocytes.

Therefore, this study aimed to investigate the effects of O. felineus ELVs (in vivo) and their associated tsRNAs (in silico) on human THP-1 monocytes. Specifically, the work included transcriptome sequencing of THP-1 monocytes following exposure to O. felineus ELVs and identification of predicted tsRNA target genes among the differentially expressed genes.

## Materials and methods

Animals. Two‑month‑old golden hamsters (Mesocricetus auratus) provided by the Animal Facility of the Institute of Cytology and Genetics SB RAS (Novosibirsk, Russia). O. felineus metacercariae were isolated from ide (Leuciscus idus) caught in the Ob River near Novosibirsk, using previously described procedures (Pakharukova et al., 2018, 2019). Each hamster was infected orally with 75 metacercariae. Adult flukes were recovered from the bile ducts two months post‑infection and washed 10 times in sterile physiological saline, as described previously. All animal experiments were approved by the Bioethics Committee of the Institute of Cytology and Genetics SB RAS (protocol No. 42, May 25, 2018).

Isolation of exosome‑like vesicles. Exosome‑like vesicles (ELVs) were isolated from the culture medium of 300 adult O. felineus flukes (RPMI‑1640 medium supplemented with 1 % glucose, 100 U/mL penicillin G, 100 μg/mL streptomy cin, and 0.25 μg/mL amphotericin B) as described previously (Pakharukova et al, 2023).

Prediction of target genes for O. felineus tRNA fragments. Previously characterised tRNA‑derived small RNAs from O. felineus ELVs were used in this study (Lishai, Pakharukova, 2026). These tsRNAs were identified by sequencing small RNA libraries generated from O. felineus ELVs. Small RNA libraries were mapped to predicted tRNA genes in the O. felineus genome (assembly GCA_004794785.1) using tRNAscan‑SE 2.0 (v.2.0.9) (Chan et al, 2021). Among 4,523 predicted tRNA loci, 4,276 pseudogenes were removed, leaving 247 sequences classified as bona fide tRNA genes. For each tRNA isotype, the most abundant tRNA sequence was selected. This yielded 50 representative tRNA sequences, which were then used as references for re‑mapping the small RNA libraries to define the most abundant tsRNA fragments (see the Table).

**Table 1.**
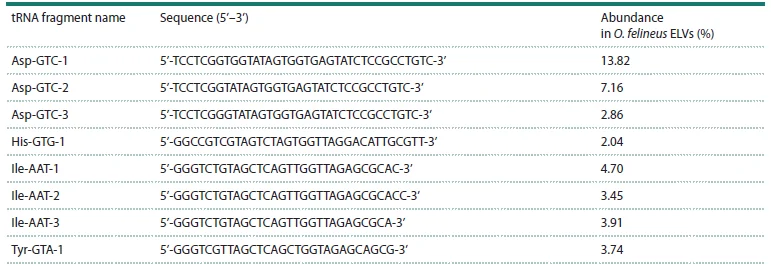
O. felineus tRNA derived fragments and their relative abundance in O. felineus ELVs

Target prediction for tsRNAs was performed using tRFtarget 2.0 (Ningshan et al, 2024) (http://trftarget.net/), which combines RNAhybrid (Rehmsmeier et al, 2004) and IntaRNA (Mann et al, 2017) and reports binding sites predicted by both algorithms with a minimum free energy below –15 kcal/mol. Human 3ʹ untranslated regions (3ʹUTRs) of protein‑coding genes were retrieved from Ensembl Genes 115 (Human genes, GRCh38.p14) (https://www.ensembl.org/ biomart/martview/330951e98fb906b5999fa97914e6118c).

Transcripts shorter than seven nucleotides or with undefined sequence were excluded. The final dataset contained 196,902 transcripts (89 % of the initial 220,194) corresponding to 19,082 genes.

For functional enrichment analysis in human monocytes, genes not expressed in monocytes or blood cells were removed using expression data from the Human Protein Atlas (https://www.proteinatlas.org/humanproteome/single+cell/ immune+cell/monocytes). The list of excluded genes is provided in Table S11. Enrichment analyses were performed using the R packages msigdb v.1.18.0 (Bhuva et al, 2025) (Gene Ontology (GO) and MSigDB curated collections (MsigDB_Curated)) and clusterProfiler v.4.18.4 (Yu et al, 2012). Visualisations were generated using ggplot2 v.4.0.1 (https://ggplot2. tidyverse.org/), LibreOffice Calc v.24.2.7.2 (https://www. libreoffice.org/discover/calc/), and Inkscape v.1.4.2 (https:// inkscape.org/ru/).

Supplementary Materials are available in the online version of the paper: https://vavilov.elpub.ru/jour/manager/files/Suppl_Lishai_Engl_30_5.xlsx


cDNA library preparation and RNA sequencing. Human THP‑1 monocytes were cultured in RPMI‑1640 medium (Gibco, USA) supplemented with 10 % fetal bovine serum (FBS; Gibco, USA), 100 U/mL penicillin (Sigma Aldrich, USA), 10 μg/mL streptomycin (Sigma Aldrich, USA), and 29.2 μg/mL L‑glutamine (Gibco, USA). To generate macrophage‑like cells, THP‑1 monocytes were treated with 100 ng/ ml phorbol 12‑myristate 13‑acetate (PMA; Solarbio, China) for 24 h, followed by a 24‑h rest period in PMA‑free medium. Total RNA was extracted from untreated cells and from cells exposed to O. felineus ELVs (10 μg/mL, 24 h) using the Aurum Total RNA Extraction Kit (Bio‑Rad, USA), according to the manufacturer’s protocol. RNA integrity and concentration were assessed using an Agilent 2100 Bioanalyzer with the Total RNA Nano Kit (Agilent Technologies, USA).

Seven cDNA libraries were prepared in total: four from untreated THP‑1 cells and three from THP‑1 cells treated with O. felineus ELVs. Libraries were constructed using the NEBNext Poly(A) mRNA Magnetic Isolation Module and the NEBNext Ultra II Directional RNA Library Prep Kit for Illumina (New England Biolabs, USA), following the manufacturer’s instructions. Size selection was performed using Agencourt AMPure XP beads (Beckman Coulter, USA). After PCR enrichment and quality control, libraries were sequenced on a DNBseq platform (BGI, China) using 2 × 150 bp paired‑end reads. Library quality was evaluated using FastQC v.0.12.1 (https://www.bioinformatics.babraham. ac.uk/projects/fastqc/). Sequencing data are available in the NCBI BioProject repository under accession PRJNA1429255.

Bioinformatic analysis of transcriptome data. Reads were aligned to the human reference genome GRCh38.p14 (GCA_000001405.29) using STAR v.2.7.10a with default parameters (Dobin et al, 2013).

Differential gene expression between THP‑1 cells treated with ELVs and untreated controls was analysed using the Wald test implemented in DESeq2 v.1.50.2. (Love et al., 2014). Genes were considered differentially expressed if they showed an absolute fold change greater than 2 and a Benjamini– Hochberg- adjusted p‑value <0.05. Low‑abundance genes with a total count <24 across all libraries were removed prior to analysis.

Variance‑stabilising transformation was applied to the count matrix using the vst() function (blind = FALSE) in DESeq2. Principal component analysis (PCA) was then performed on the transformed expression matrix using the prcomp() function in the R stats package v.4.5.2.

Pathway enrichment analysis was performed using the Kyoto Encyclopedia of Genes and Genomes (KEGG) and Molecular Signatures Database (MSigDB) collections. KEGG annotations were obtained via org.Hs.eg.db v.3.22.0, and enrichment analyses were carried out with clusterProfiler v.4.18.4 and msigdb v.1.18.0. Results were visualised using LibreOffice Calc v.24.2.7.2 and Inkscape v.1.4.2. Functional enrichment for differentially expressed genes that were also predicted tsRNA targets was performed using the same pipeline.

To assess whether tsRNA target genes were significantly enriched among differentially expressed genes in THP‑1 monocytes, a Pearson chi‑square test with Yates’ continuity correction was applied using the chisq.test() function from the R stats package v.4.5.2.

Interaction networks between tsRNA fragments and differentially expressed genes were constructed and manually curated in Inkscape v.1.4.2.

## Results

**O. felineus exosome‑like vesicles alter gene expression and activate immune‑ and cell cycle-related pathways in THP‑1 monocytes**

On average, 43.8 million paired‑end reads were obtained per RNA‑seq library. Mapping to the human reference genome yielded approximately 37 million uniquely mapped reads per library (84.63 %), while an additional 3.7 million reads per library (8.63 %) mapped to multiple genomic locations.

Principal component analysis revealed clear clustering of samples according to treatment status, separating ELV‑treated from untreated THP‑1 cells (Fig. 1a). A total of 1,484 genes were identified as differentially expressed (absolute fold change >1.5, adjusted p-value <0.05), including 392 downregu-lated and 1,092 upregulated genes (Fig. 1b; Table S2). Among the most strongly upregulated genes (log2 fold change >4.6; >25‑fold increase) were JCHAIN, LINC03063, CD8B2, MLC1, and RNASE2. In contrast, the expression of LOC124902900, HBA1, HBA2, LOC124900173, and LOC112268061 was downregulated more than 6.9‑fold (log2 fold change < –2.78).

**Fig. 1.**
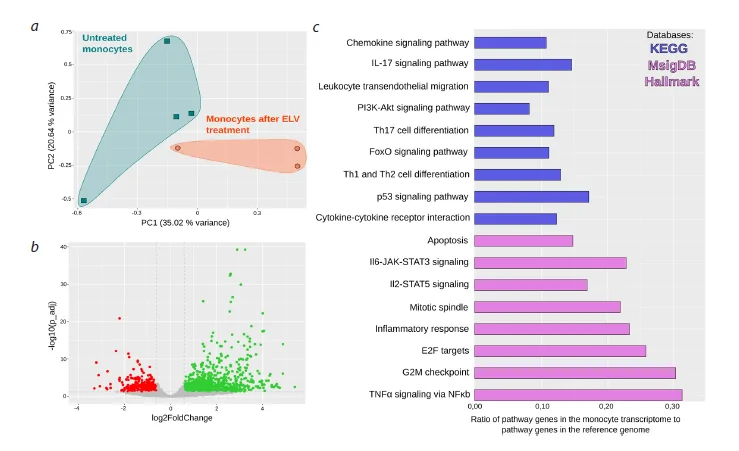
Transcriptomic profile of THP-1 monocytes treated with O. felineus exosome-like vesicles а – principal component analysis (PCA) of RNA seq data showing separation of samples according to treatment with O. felineus exosome-like vesicles (ELVs) versus untreated THP-1 monocytes; b – Volcano plot of differentially expressed genes (DEGs) in ELV treated cells compared with untreated monocytes. Genes with adjusted p value < 0.05 (−log10(padj) > 1.3) and absolute fold change > 1.5 are highlighted: upregulated genes (log2 fold change > 0.6) are shown in green and downregulated genes (log2 fold change < −0.6) in red; c – functional enrichment analysis of DEGs using the Kyoto Encyclopedia of Genes and Genomes (KEGG) and the Hallmark gene sets from the Molecular Signatures Database (MSigDB).

Functional enrichment analysis of differentially expressed genes was performed using KEGG and the Hallmark collection from MSigDB. Among upregulated genes, 43 KEGG pathways were significantly enriched (adjusted p < 0.05), whereas nine KEGG pathways were enriched among downregulated genes. For the Hallmark gene sets, eight pathways were significantly enriched in the upregulated gene set and one pathway in the downregulated gene set (adjusted p < 0.05). Full pathway lists and statistics are provided in Table S2.

Among the upregulated DEGs, enrichment was observed for pathways related to immune processes, including leukocyte transendothelial migration (hsa04670), Th17 cell differentiation (hsa04659), Th1 and Th2 cell differentiation (hsa04658), and inflammatory response (M5932). Pathways associated with cell cycle regulation and cell death were also enriched, such as mitotic spindle assembly (M5893), the G2/M checkpoint (M5901), apoptosis (M5902), and the p53 signalling pathway (hsa04115) (Fig. 1c).

**Prediction of O. felineus tsRNA target genes among genes expressed in human monocytes**

For target prediction, 3ʹ untranslated region (3ʹUTR) sequences of human protein‑coding genes were used (196,902 3ʹUTRs corresponding to 19,082 genes). Genes were retained for further analysis if at least one of their 3ʹUTR transcripts contained a predicted binding site for at least one O. felineus tRNA‑derived fragment. This yielded 2,145 target genes among human protein‑coding genes. Because the focus of this study was the potential impact of O. felineus tsRNAs on human monocytes, genes not expressed in blood cells or monocytes were removed from the target list. After this filtering, 1,299 genes remained as potential targets of O. felineus tsRNAs in human monocytes. The largest number of predicted targets was found for the tsRNA Asp‑GTC‑3 (522 genes), whereas the smallest number was observed for His‑GTG‑1 (95 genes) (Fig. 2a; Table S3).

**Fig. 2.**
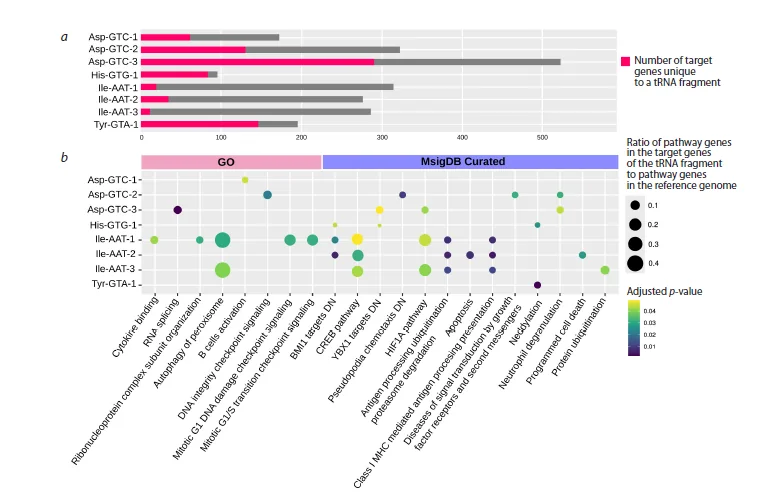
Predicted O. felineus tsRNA target genes among transcripts expressed in human monocytes а – number of predicted target genes for each O. felineus tRNA derived fragment. Genes with a binding site for only a single tsRNA are shown in pink, whereas genes targeted by multiple tsRNAs are shown in grey; b – functional enrichment of predicted target genes for each tsRNA using Gene Ontology (GO) and the curated collection of the Molecular Signatures Database (curated MSigDB).

The largest number of enriched pathways was observed for target genes of the tsRNA Ile‑AAT‑1: 15 Hallmark/curated MSigDB gene sets and 26 Gene Ontology (GO) terms. In contrast, target genes of Asp‑GTC‑1 and Tyr‑GTA‑1 showed enrichment for the smallest number of pathways (two GO terms for 172 Asp‑GTC‑1 targets and two MSigDB pathways for 195 Tyr‑GTA‑1 targets). Notably, the largest target set (Asp‑GTC‑3) and one of the smallest sets (His‑GTG‑1) were associated with a similar number of enriched pathways (18 for Asp‑GTC‑3 and 17 for His‑GTG‑1).

In human monocytes, tsRNA target genes were enriched for pathways linked to cell cycle control and cell death, including cell cycle regulation (M16357, GO:0044774, GO:0044819) and programmed cell death (M27436, M15303). Additional enrichment was observed for immune and inflammatory processes, such as neutrophil degranulation (M27620), antigen presentation via MHC class I (M1066), proteasome‑mediated antigen processing (M1070), B‑cell activation (M10657), and cytokine binding (M18255). These data are summarised in Table S3

Taken together, these results indicate that O. felineus ELV‑derived tsRNAs have predicted binding sites in host genes whose products participate in key cellular processes. By modulating the expression of such genes through tsRNA– mRNA interactions, liver flukes may be able to influence the activity and functional state of host immune cells.

**Prediction of tsRNA target genes among DEGs in THP‑1 monocytes**

Although THP‑1 cells are a widely used model for studying monocyte‑mediated immune responses, substantial transcriptional differences exist between primary human monocytes and THP‑1 cells (Bosshart, Heinzelmann, 2016; Liu Y. et al., 2021). Therefore, when assessing the overlap between O. felineus tsRNA targets and differentially expressed genes in ELV‑treated THP‑1 monocytes, all 2,145 predicted tsRNA target genes were considered, rather than only those verified as expressed in monocytes. A significant enrichment of tsRNA target genes was observed among DEGs (Pearson chi‑square test with Yates’ correction = 6.145, p = 0.014). Of the 1,484 DEGs identified in THP‑1 cells after ELV treatment, 159 genes were predicted targets of O. felineus tsRNAs. These genes were used to construct a tsRNA–mRNA interaction network (Fig. 3; Table S4).

**Fig. 3.**
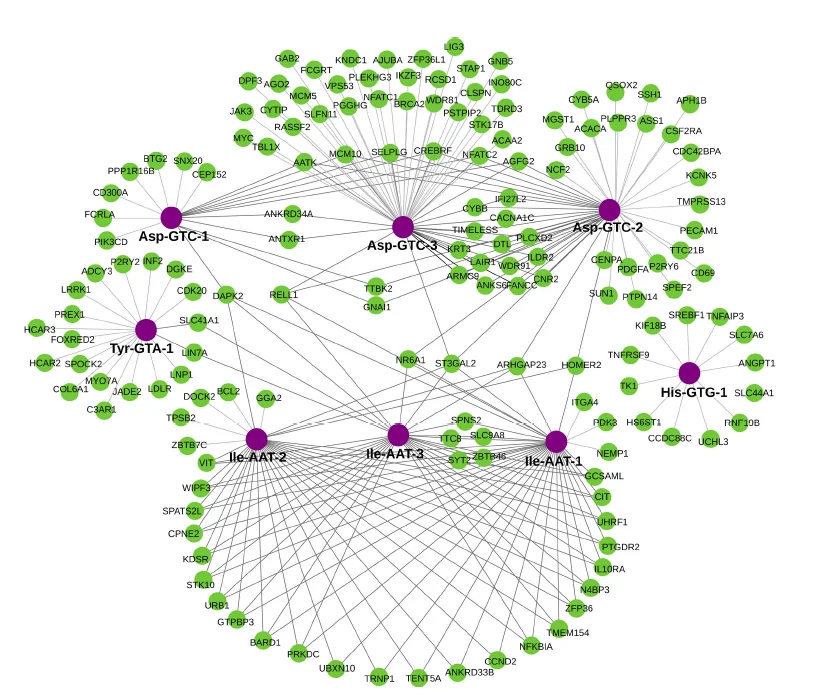
Interaction network between O. felineus tsRNAs and differentially expressed genes in THP-1 monocytes.

Genes whose expression was significantly altered in THP‑1 monocytes following treatment with O. felineus exosome‑like vesicles and that were also predicted tsRNA targets were mapped onto a tsRNA–mRNA interaction network. These target genes were enriched for pathways involved in cell cycle control and cell death, including DNA integrity checkpoint (M16357), G1/S transition (M2074), apoptosis (M15303), and programmed cell death (M27436), as well as immune response pathways such as the monocyte pathway (M4956) mononuclear macrophage differentiation (M25144), antigen presentation via MHC class I (M1066), and proteasome‑mediated antigen processing (M1070). Detailed gene and pathway annotations are provided in Table S4.

## Discussion

Exosomes and exosome-like vesicles (EVs) are key and evolutionarily conserved mediators of intercellular communication in many organisms, yet their role in parasite–host interactions has only recently begun to be systematically explored. EVs contain diverse bioactive molecules, including proteins, small and long non‑coding RNAs, mRNAs, and lipids (Zhang et al., 2022; Pakharukova et al., 2023).

Parasite‑derived exosomes can be internalised by host cells and alter both gene expression and the secretion of effector proteins. For example, exosomes from Trichomonas vaginalis stimulated increased production of the pro‑inflammatory cytokines IL‑6 and IL‑8 in human cervical epithelial cells Ect1 E6/E7 (Twu et al., 2013). Mouse RAW264.7 macrophages internalised exosomes from Toxoplasma gondii, leading to increased secretion of IL‑12, TNFα and IFNγ together with reduced production of the anti‑inflammatory cytokine IL‑10 (Li Y. et al., 2018).

In Schistosoma japonicum, exosomes modulated the expression of genes involved in Rap1, TNF and Toll‑like receptor signalling as well as cytokine–cytokine receptor interaction pathways. Treatment of RAW264.7 macrophages with exosomal microRNAs enhanced TNFα production (Liu J. et al., 2019). In line with these findings, treatment of PMA‑differentiated human THP‑1 monocytes with O. felineus exosome‑like vesicles (ELVs) in the present study resulted in increased expression of genes associated with TNFα signalling and pro‑inflammatory responses, including TNF, CCL2, CXCL2, CXCL3, IL6ST, NFKB2, REL, and RELB.

Interestingly, exosomes from Fasciola hepatica did not enhance TNFα production in THP‑1 monocytes but instead increased the levels of anti‑inflammatory mediators such as TGF‑β and NOS2 (Sánchez‑López et al., 2023). These divergent responses to ELVs from F. hepatica versus O. felineus are consistent with studies showing that excretory–secretory products and secreted proteins from F. hepatica drive macrophages towards an anti‑inflammatory M2 phenotype (Flynn et al., 2007; Figueroa‑Santiago, Espino, 2014), whereas exposure to adult Opisthorchis viverrini and excretory–secretory products from Clonorchis sinensis – species closely related to O. felineus – promotes a pro‑inflammatory M1 phenotype (Hongsrichan et al., 2014; Kim et al., 2017). However, the specific molecular components within the excretory–secretory products of these trematodes that account for the distinct host immune responses remain unknown.

tRNA‑derived small RNAs (tsRNAs) represent a distinct class of small non‑coding RNAs and have been identified in exosomes from several parasitic species (Fontenla et al., 2022; Kusakisako et al., 2023; Natali et al., 2023). Nevertheless, their roles in parasite–host communication remain poorly characterised. To date, there are no experimental data directly demonstrating the effects of parasite‑derived tsRNAs from exosomes on host cells in vivo or in vitro.

At the same time, tsRNAs are actively studied as disease biomarkers and as novel regulatory molecules, including in immune cells. In colorectal cancer, elevated levels of specific tsRNAs (tRF‑3022b, tRF‑3030b and tRF‑5008b) have been detected in plasma exosomes, and reducing the levels of these fragments induces cell‑cycle arrest and apoptosis in tumor cells; tRF‑3022b can also reduce M2 macrophage polarisation by binding to macrophage migration inhibitory factor (MIF) (Lu et al., 2022). In THP‑1 cells, the 5ʹ‑His‑GUG tsRNA is elevated in monocytes and decreases upon differentiation into macrophages, and macrophages transfected with this fragment exhibit reduced survival following exposure to apoptotic stimuli (Jayaram et al., 2025).

Parasite‑derived tsRNAs from O. felineus ELVs possess predicted binding sites within the 3ʹUTRs of human genes, although their functional impact is likely to be cell‑type specific. In human monocytes, the predicted tsRNA targets identified in this study were enriched for pathways related to cell cycle control, cell death, and immune responses. These observations suggest that trematodes may influence such processes in host immune cells by secreting ELVs containing tsRNAs

To assess the potential impact of O. felineus tsRNAs on the expression of their target genes, tsRNA targets were intersected with DEGs in THP‑1 monocytes after ELV treatment, revealing significant enrichment. For some predicted targets, expression levels decreased, whereas others were upregulated. This pattern may reflect non‑canonical modes of gene regulation analogous to those described for microRNAs, which can also enhance gene expression under certain conditions (Valinezhad et al., 2014).

Target genes whose expression changed significantly in ELV‑treated THP‑1 monocytes were associated with cell‑cycle and immune pathways, which substantially overlapped with pathways enriched among all tsRNA targets expressed in monocytes and blood cells. Together, these findings support the idea that parasite‑derived tsRNAs may contribute to the regulation of these processes in host monocytes by modulating the expression of a subset of genes.

Further functional studies are required to confirm the involvement of parasite tsRNAs in regulating these pathways. In particular, in vivo experiments using synthetic tsRNA analogues will be necessary to validate their direct effects on host gene expression and immune cell function. Moreover, because O. felineus excretory–secretory products and exosome‑like vesicles contain a broad spectrum of bioactive molecules (Pakharukova et al., 2023), synergistic effects of multiple components on host cells are likely. Thus, it will be important to investigate other classes of regulatory molecules present in O. felineus ELVs, including microRNAs, to obtain a comprehensive view of how this parasite modulates the host immune response.

## Conclusion

The role of exosome‑like vesicles (ELVs) released by the liver fluke Opisthorchis felineus in shaping the host immune response remains poorly understood. In particular, the contribution of small non‑coding RNAs carried by these vesicles, such as tRNA‑derived fragments (tsRNAs), has not been characterised in detail, even though tsRNAs are now actively investigated as disease biomarkers and as a novel class of regulatory molecules

In this study, transcriptomic profiling of human THP‑1 monocytes treated with O. felineus ELVs was combined with in silico prediction of O. felineus tsRNA target genes among transcripts expressed in human monocytes and with the identification of tsRNA targets within the set of differentially expressed genes in ELV‑treated THP‑1 cells.

Further validation in vivo is required to confirm these findings. Future studies are necessary to be focused on: 1) assessment of proliferation, migration and cell‑cycle dynamics in host cells exposed to synthetic analogues of O. felineus tsRNAs; 2) verification of predicted target tsRNA binding sites using reporter system; and 3) quantification of target gene expression at both the mRNA level (quantitative real‑time PCR) and the protein level (Western blot analysis).

## Conflict of interest

The authors declare no conflict of interest.
